# Supplementary material for: Metabolic glycan labeling immobilizes dendritic cell membrane and enhances antitumor efficacy of dendritic cell vaccine
Source: Nat Commun. 2023 Aug 19;14:5049. doi: 10.1038/s41467-023-40886-7 (PMC10439884; doi:10.1038/s41467-023-40886-7)
Supplement: Supplementary file 1 — Supplementary Information [file 41467_2023_40886_MOESM1_ESM.pdf]

Metabolic Glycan Labeling Immobilizes Dendritic Cell Membrane and Enhances Antitumor Efficacy of Dendritic Cell Vaccine

Joonsu Han[1], Rimsha Bhatta[1], Yusheng Liu[1], Yang Bo[1], Alberto Elosegui Artola[2], Hua Wang[1,3,4,5,6,7,8*]

[1]Department of Materials Science and Engineering, University of Illinois at Urbana-Champaign, Urbana, IL 61801, USA. [2]The Francis Crick Institute, United Kingdom. [3]Cancer Center at Illinois (CCIL), Urbana, IL 61801, USA. [4]Department of Bioengineering, University of Illinois at Urbana-Champaign, Urbana, IL 61801, USA. [5]Carle College of Medicine, University of Illinois at Urbana-Champaign, Urbana, IL 61801, USA. [6]Beckman Institute for Advanced Science and Technology, University of Illinois at Urbana-Champaign, Urbana, IL 61801, USA. [7]Materials Research Laboratory, University of Illinois at Urbana-Champaign, Urbana, IL 61801, USA. [8]Institute for Genomic Biology, University of Illinois at Urbana-Champaign, Urbana, IL 61801, USA.

*correspondence should be addressed to huawang3@illinois.edu

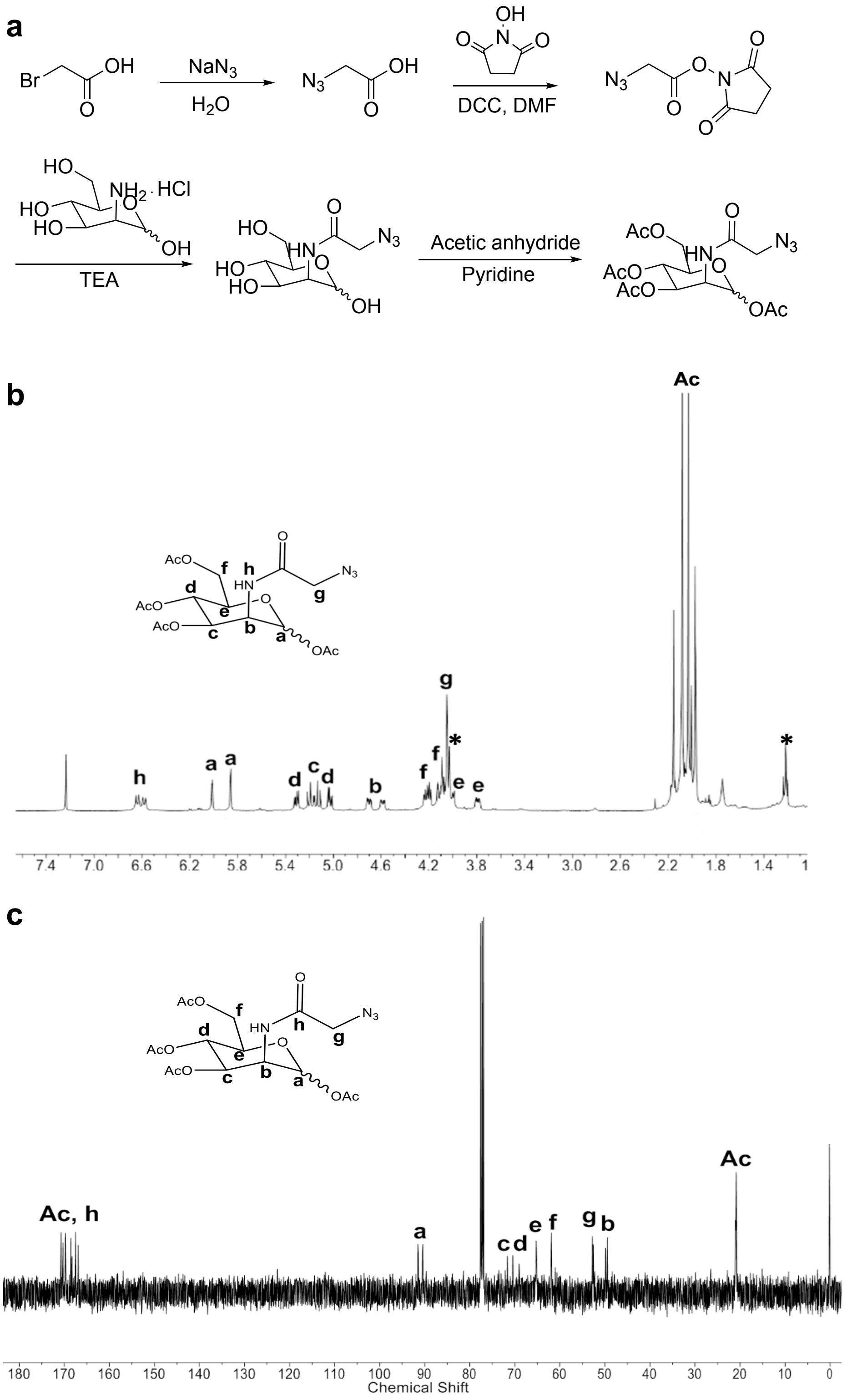

Supplementary Figure 1. Synthesis and characterization of Ac_4ManNAz. (a) Synthetic route of Ac_4ManNAz. (b) ^{1}H NMR spectrum of Ac_4ManNAz in chloroform. (c) ^{13}C NMR spectrum of Ac_4ManNAz in chloroform.

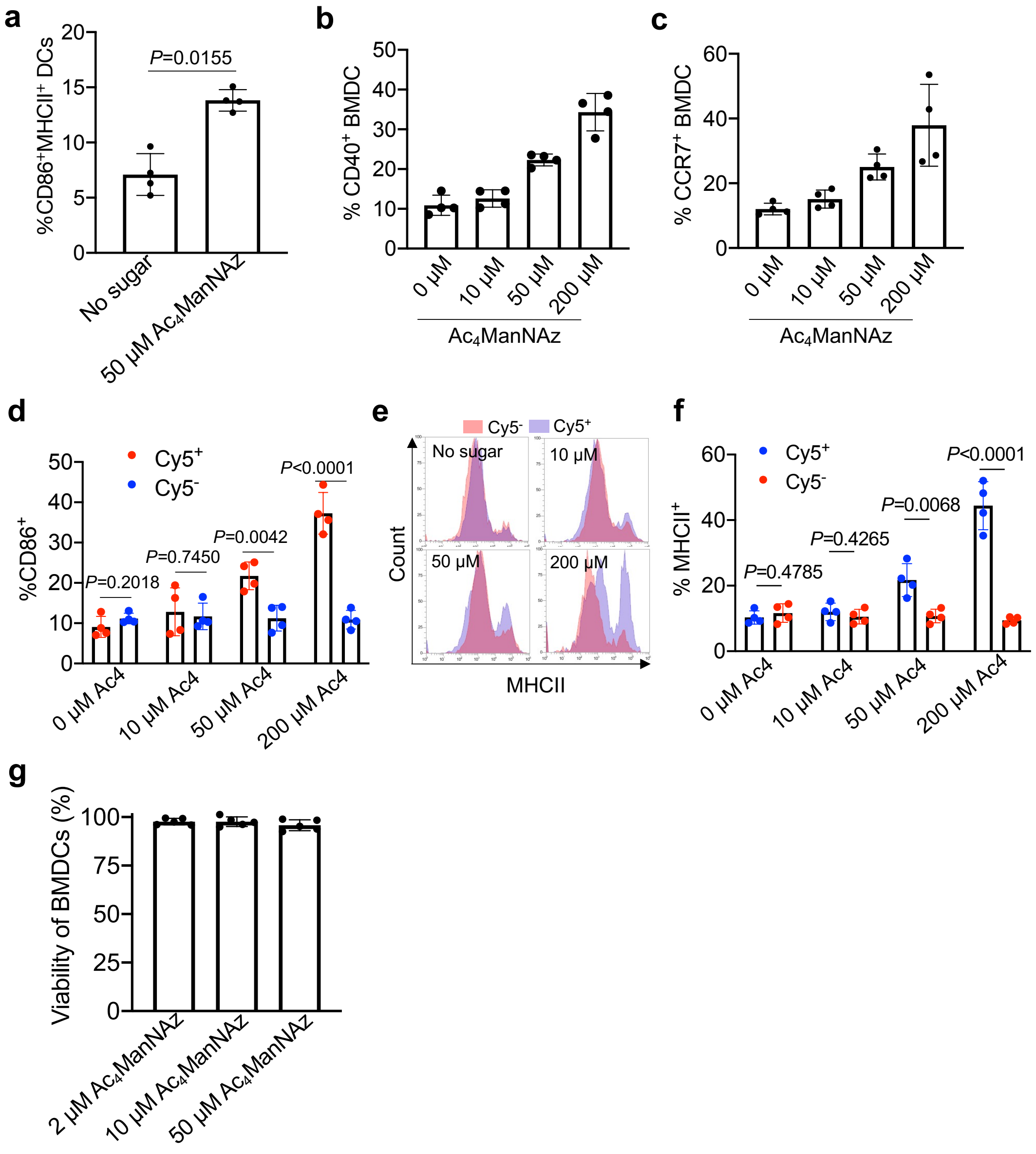

Supplementary Figure 2. Metabolic glycan labeling improves the activation status of DCs. Percentages of (a) $CD86^+MHCII^+$ DCs, (b) $CD40^+$ DCs, and (c) $CCR7^+$ DCs after 3-day incubation with azido-sugars or PBS (n=4). (d) Percentages of $CD86^+$ DCs within $Cy5^+$ and $Cy5^-$ subpopulations after 3-day incubation with different concentrations of $Ac_4ManNAz$ (n=4). (e) Representative MHCII histograms of DCs within $Cy5^+$ and $Cy5^-$ subpopulations after treated with different concentrations of $Ac_4ManNAz$ for three days and stained with FITC-conjugated anti-MHCII and DBCO-Cy5. Also shown are (f) percentages of $MHCII^+$ DCs within $Cy5^+$ and $Cy5^-$ subpopulations following the same treatment as in (e) (n=4). (g) Viability of DCs after 3-day incubation with $Ac_4ManNAz$ (n=5). All the numerical data are presented as mean ± SD (for a, d, and f, two-tailed Welch's t-test was used; $0.01 < {*P} \leq 0.05$; $**P \leq 0.01$; $***P \leq 0.001$).

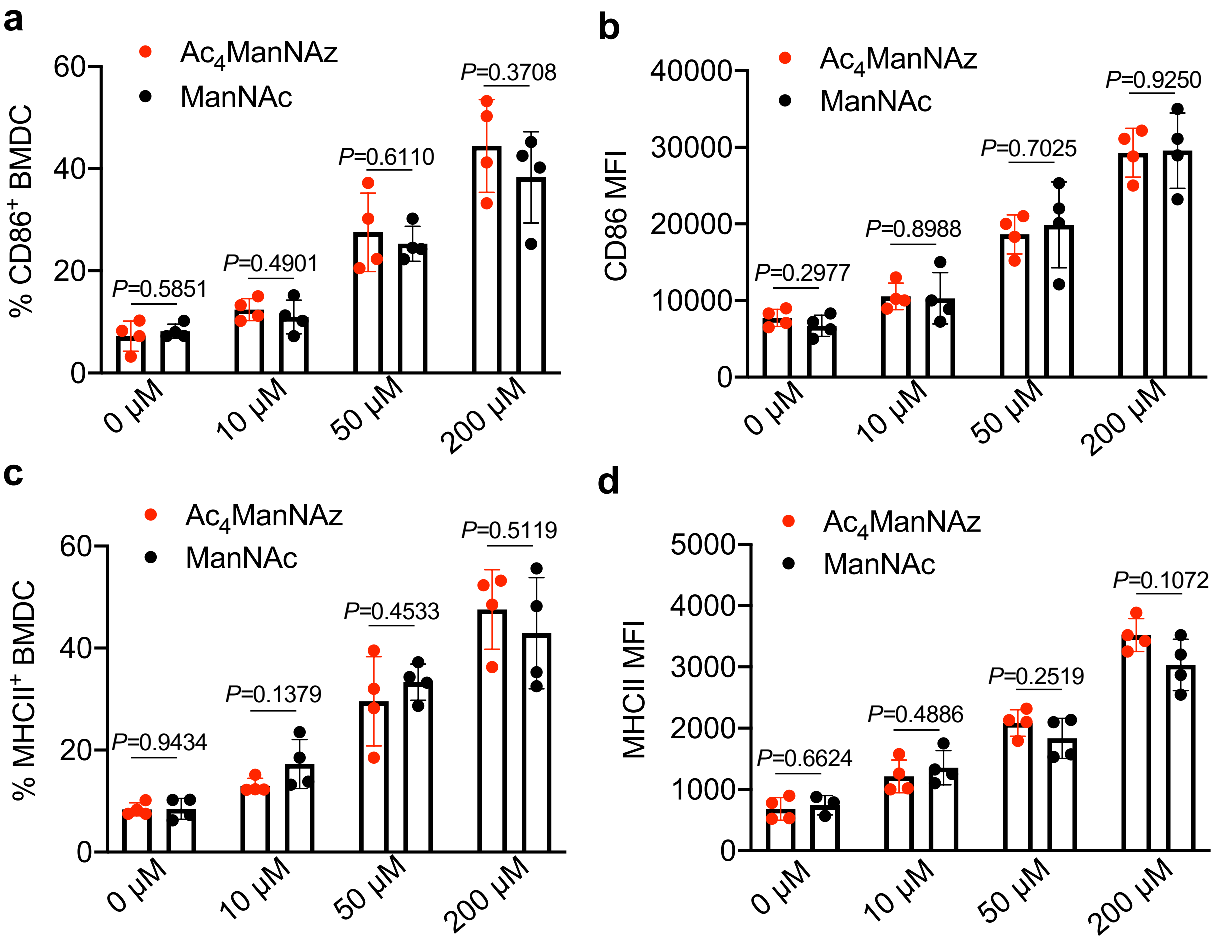

Supplementary Figure 3. Azido tag itself does not further induce the activation of DCs. (a) Percentages of CD86+ DCs and (b) mean CD86 fluorescence intensity of DCs after 3-day incubation with $Ac_4ManNAz$ or ManNAc (n=4). Also shown are (c) percentages of MHCII+ DCs and (d) mean MHCII fluorescence intensity of DCs after 3-day incubation with $Ac_4ManNAz$ or ManNAc (n=4). All the numerical data are presented as mean ± SD (for a-d, two-tailed Welch's t-test was used; 0.01 < **P* ≤ 0.05; ***P* ≤ 0.01; ****P* ≤ 0.001).

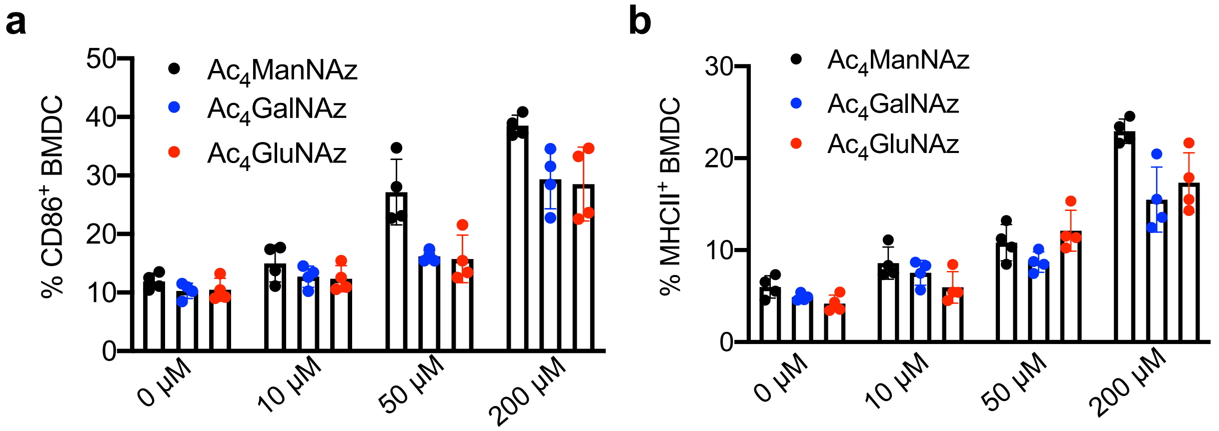

Supplementary Figure 4. DC activation effect of different types of azido-sugars. (a) Percentages of CD86$^+$ DCs after 3-day incubation with different sugars (n=4). (b) Percentages of MHCII$^+$ DCs after 3-day incubation with different sugars (n=4). All the numerical data are presented as mean ± SD.

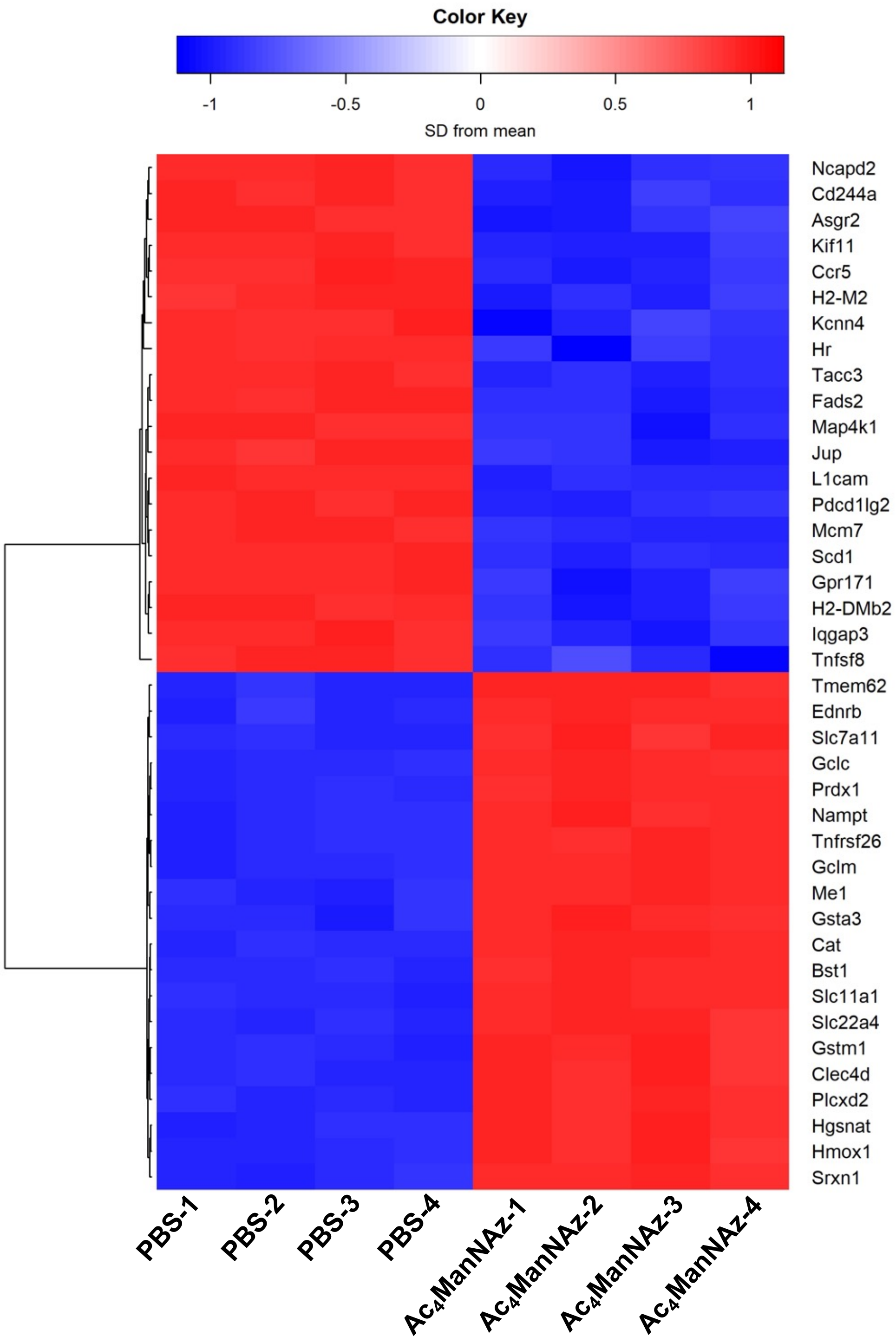

Supplementary Figure 5. Heatmap of 20 most significant up and down regulated genes from DCs treated with $Ac_4ManNAz$ (200 µM) or PBS for three days.

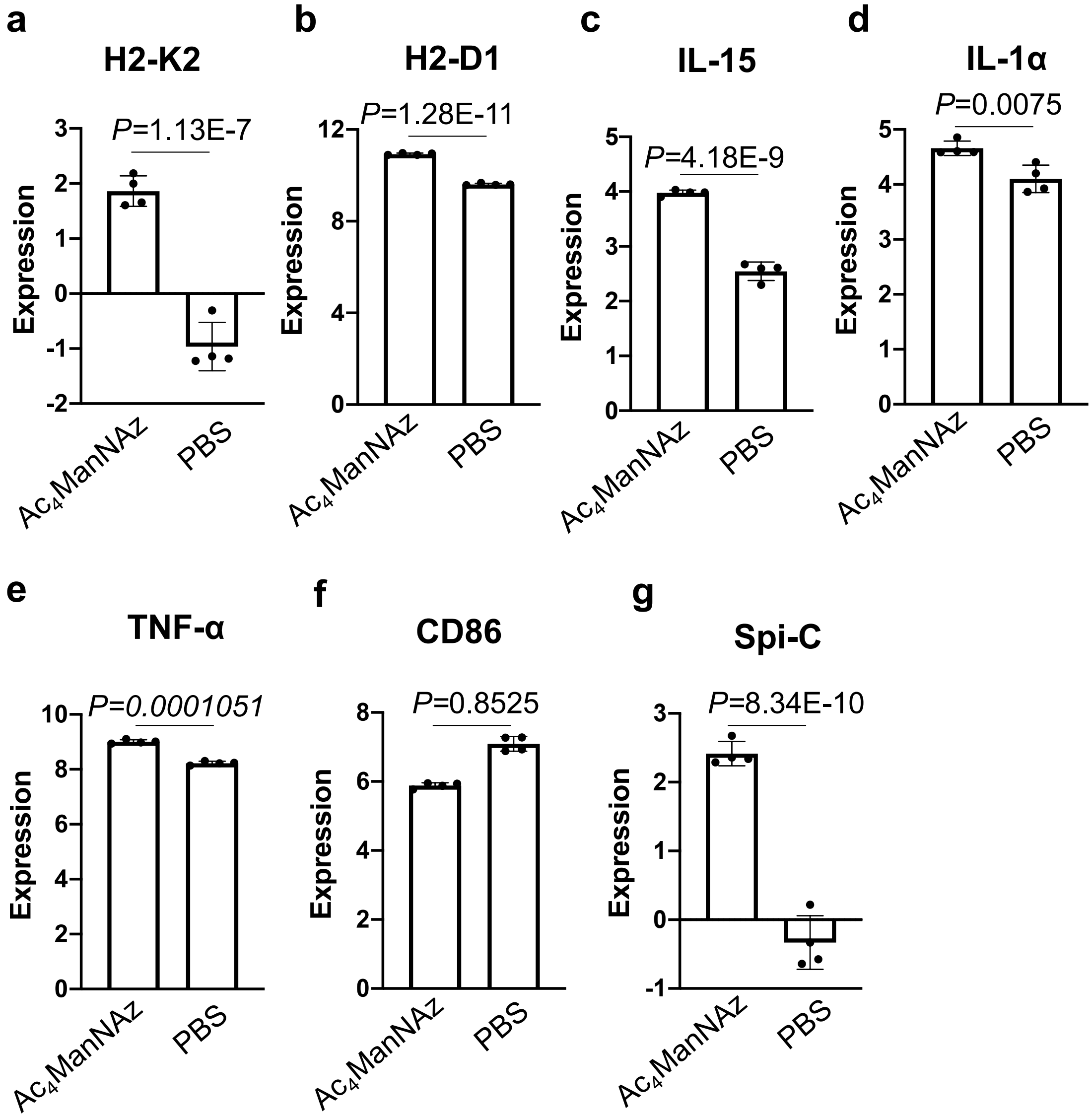

Supplementary Figure 6. Metabolic glycan labeling radically alters the gene expression of DCs. Normalized expression of (a) H2-K2 (MHCI), (b) H2-D1 (MHCI), (c) IL-15, (d) IL1α, (e) TNF-α, (f) CD86, and (g) Spi-C mRNAs, as determined by the transcriptome analysis (n=4). All the numerical data are presented as mean ± SD (two-tailed Welch's t-test was used; $0.01 < *P \leq 0.05$; $**P \leq 0.01$; $***P \leq 0.001$).

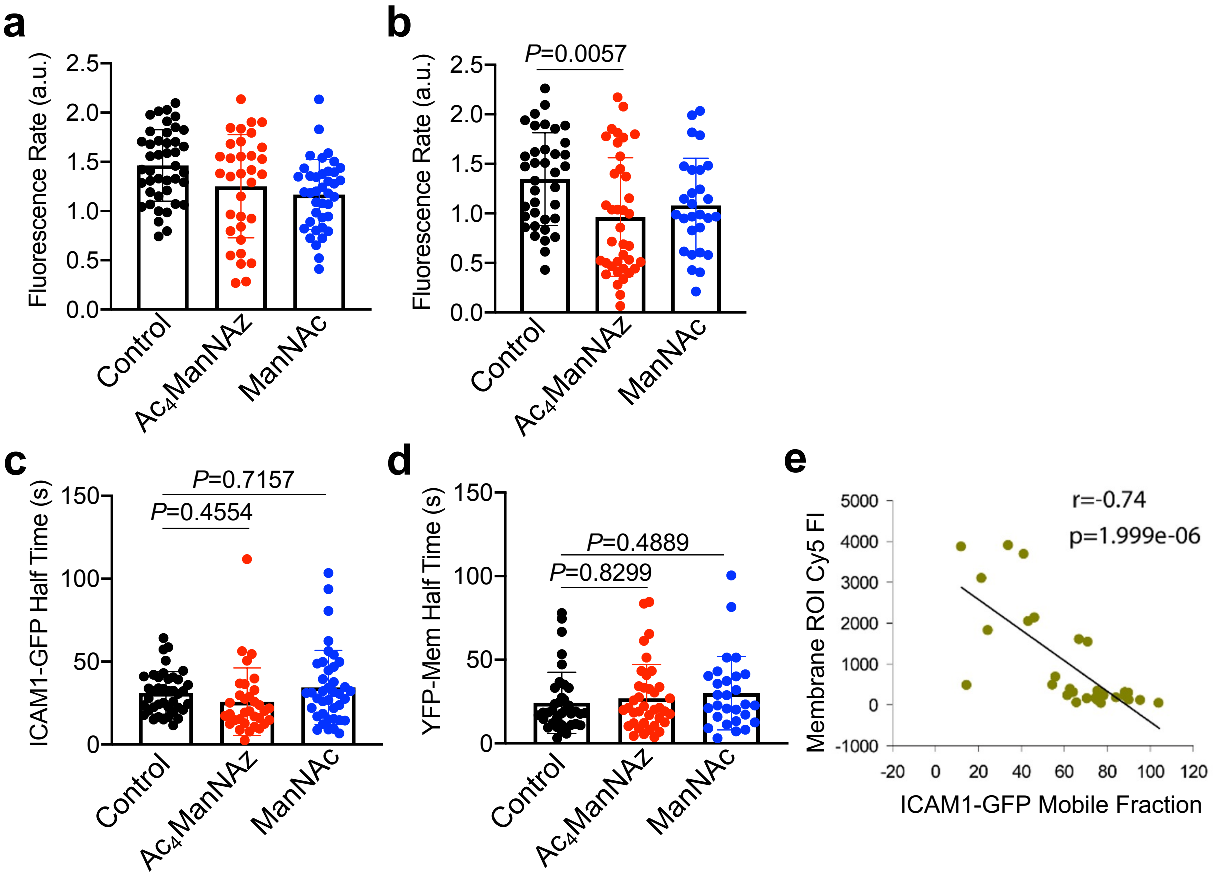

Supplementary Figure 7. Metabolic glycan labeling reduces the membrane mobility of DCs. DCs were transfected with ICAM-1-GFP or YFP-Mem, and incubated with $Ac_4ManNAz$ or ManNAc or PBS for three days. (a) ICAM-1-GFP fluorescence recovery rate of DCs after photobleaching (n>30). (b) YFP-Mem fluorescence recovery rate of DCs after photobleaching (n>30). Also shown are the (c) ICAM1-GFP and (d) YFP-Mem recovery half time of DCs during the FRAP assay (n>30). (e) Correlation between Cy5 fluorescence intensity of membrane regions of interest (ROIs) and ICAM1-GFP mobile fractions. ICAM1-GFP expressing DCs were treated with $Ac_4ManNAz$ for three days and stained with DBCO-Cy5 for 30 min before FRAP assays. All the numerical data are presented as mean ± SD (one-way ANOVA with post hoc Fisher's LSD test was used; $0.01 < *P \leq 0.05$; $**P \leq 0.01$; $***P \leq 0.001$).

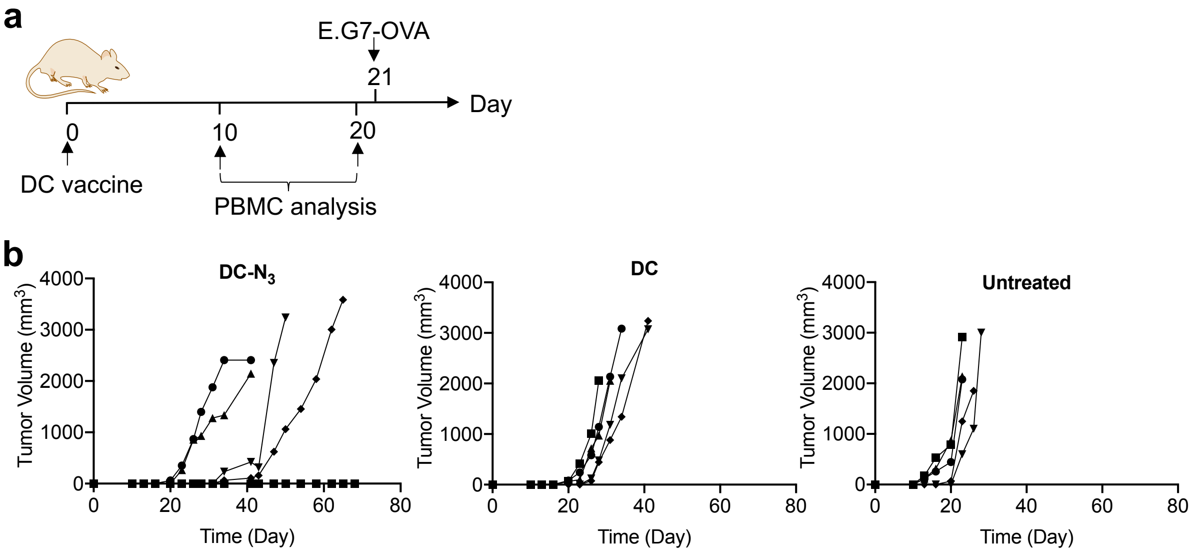

Supplementary Figure 8. Metabolic glycan labeling improves the CTL response and antitumor efficacy of DC vaccines. (a) Timeframe of the vaccination study. DCs pretreated with $Ac_4ManNAz$ or PBS for three days and pulsed with SIINFEKL peptide for 16 h were injected on day 0. E.G7-OVA tumor cells were inoculated on day 21. (b) Tumor growth curves of mice in different groups.

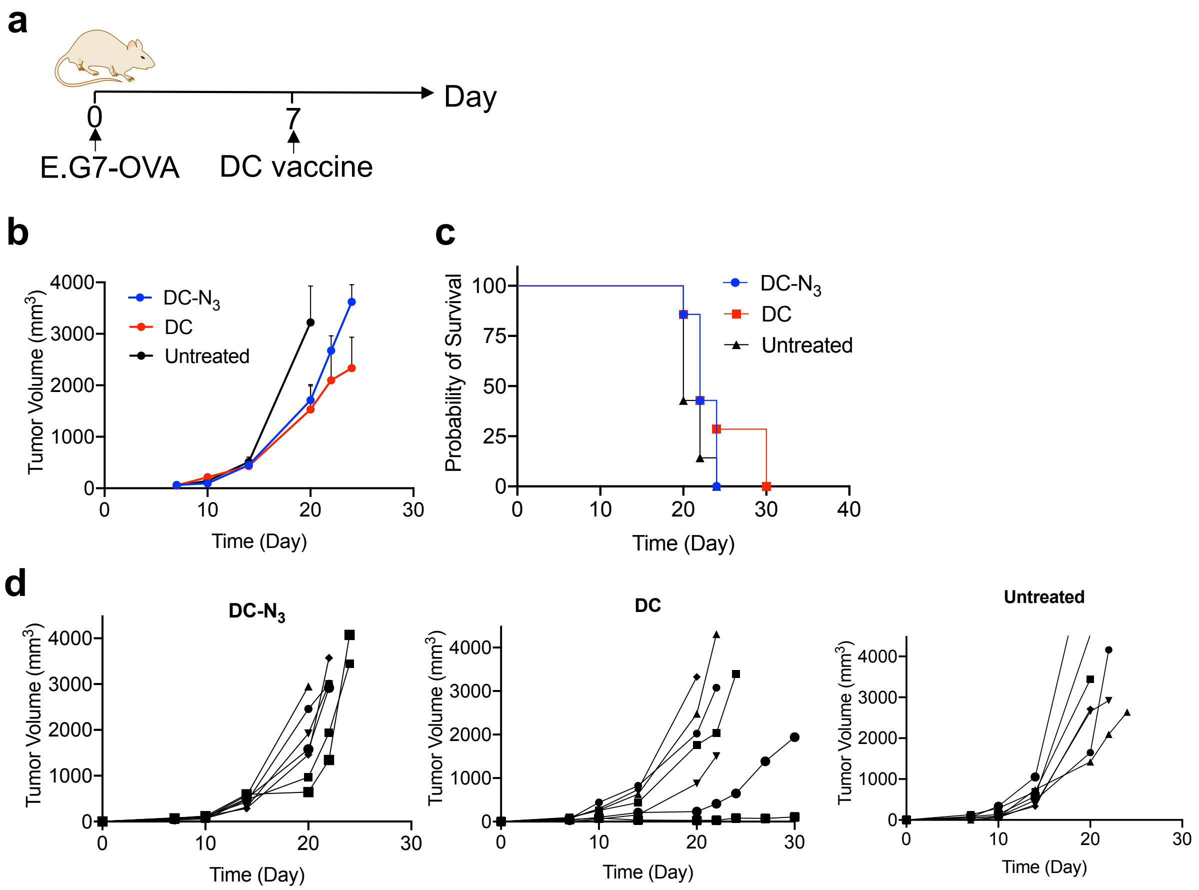

Supplementary Figure 9. A single dose of DC-N_3 vaccine fails to exert enhanced antitumor efficacy. (a) Timeframe of the tumor efficacy study. DCs were pretreated with Ac_4ManNAz for three days and pulsed with SIINFEKL peptide for 16 h. (b) Average E.G7-OVA tumor volume of each group over the course of therapeutic tumor study (n=7). (c) Kaplan-Meier plots for all groups (n=7). (d) Tumor growth curves of individual mice in different groups. The numerical data in (b) are presented as mean ± SEM.

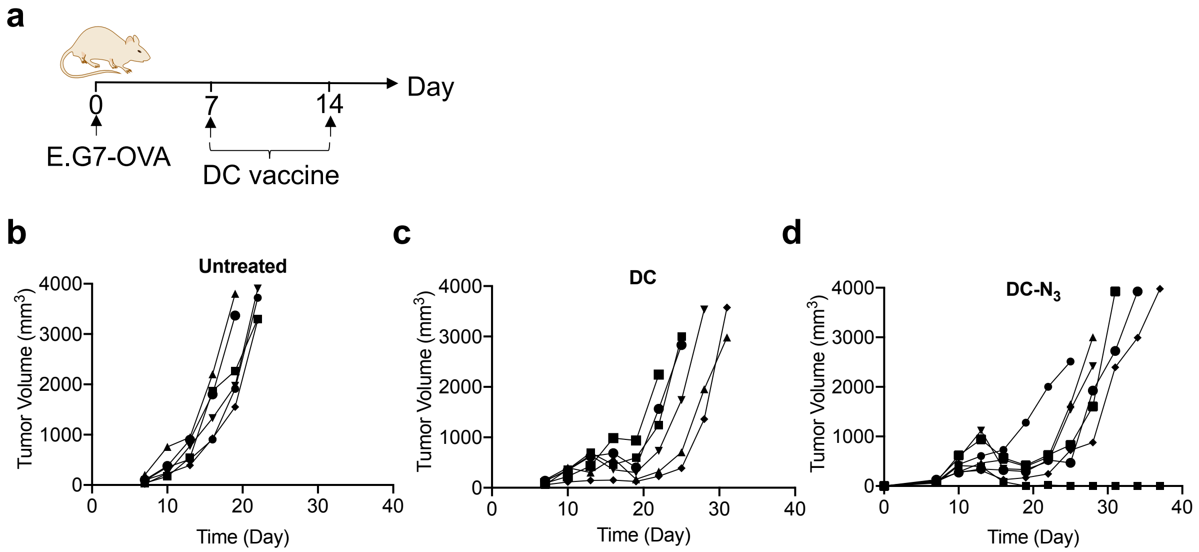

Supplementary Figure 10. Two doses of DC-N_3 vaccine exert enhanced antitumor efficacy. (a) Timeframe of the vaccination study. DCs were pretreated with $Ac_4ManNAz$ for three days and pulsed with SIINFEKL peptide for 16 h. (b-d) Tumor growth curves of individual mice in different groups.

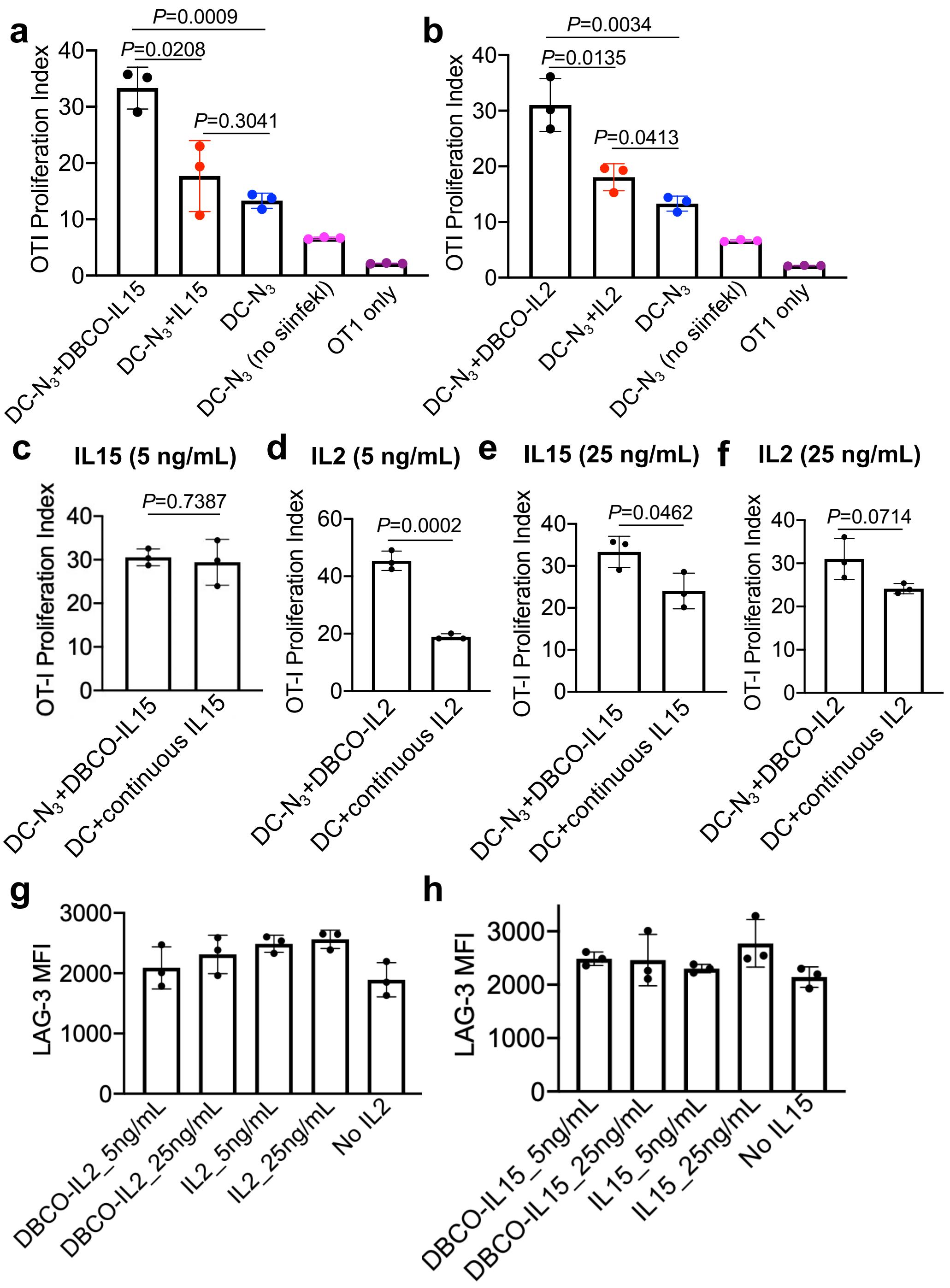

Supplementary Figure 11. Azido-labeled DCs enable conjugation of DBCO-cytokines for enhanced priming of CD8$^+$ T cells *in vitro*. (a) Proliferation index of OT-1 cells after 3-day coculture with DCs. DCs were pretreated with $Ac_4ManNAz$ or PBS for three days, pulsed with SIINFEKL peptide for 16 h, and incubated with DBCO-IL-15 or IL-15 (25 ng/mL) for 30 min. (b) Proliferation index of OT-1 cells after 3-day coculture with DCs. DCs were pretreated with $Ac_4ManNAz$ or PBS for three days, pulsed with SIINFEKL peptide for 16 h, and incubated with DBCO-IL-2 or IL-2 (25 ng/mL) for 30 min. (c,e) Proliferation index of OT-1 cells after 3-day

coculture with IL-15-conjugated DCs or control DCs + continuous IL-15 incubation. (d,f) Proliferation index of OT-1 cells after 3-day coculture with IL-2-conjugated DCs or control DCs + continuous IL-2 incubation. Also shown are the LAG-3 expression levels of OT1 cells after 3-day co-culture with DCs pre-conjugated with IL2 (g) or IL15 (h). All the numerical data are presented as mean ± SD (for a and b, one-way ANOVA with post hoc Fisher's LSD test was used; for c-f, two-tailed Welch's t-test was used; $0.01 < *P \leq 0.05$; $**P \leq 0.01$; $***P \leq 0.001$). n= 3 biologically independent samples for all experiments.

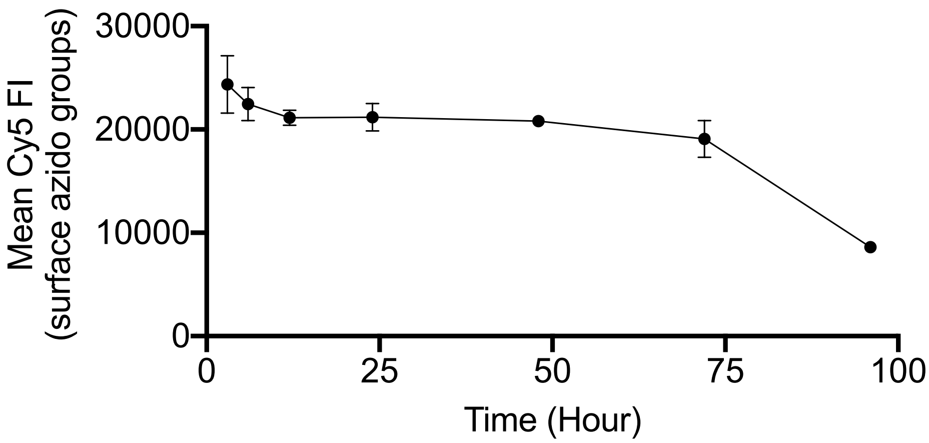

Supplementary Figure 12. Turn-over rate of cell-surface azido groups. BMDCs were pretreated with $Ac_4ManNAz$ for three days and then transferred to fresh medium, followed by the detection of cell-surface azido groups using DBCO-Cy5 at different times (n=4). All the numerical data are presented as mean ± SD.

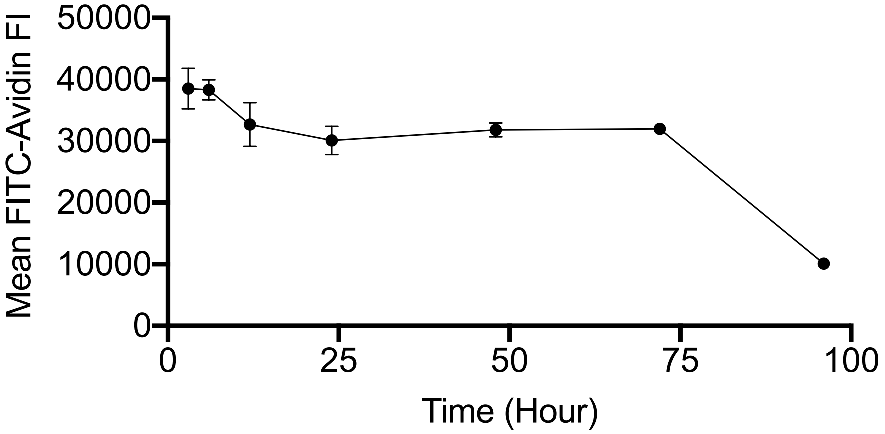

Supplementary Figure 13. Membrane retention of molecules conjugated onto DCs. BMDCs were pretreated with $Ac_4ManNAz$ for three days and then incubated with DBCO-Biotin for 30 min. DCs were then transferred to fresh media and cell-surface biotin was detected by FITC-avidin at different times (n=4). All the numerical data are presented as mean ± SD.

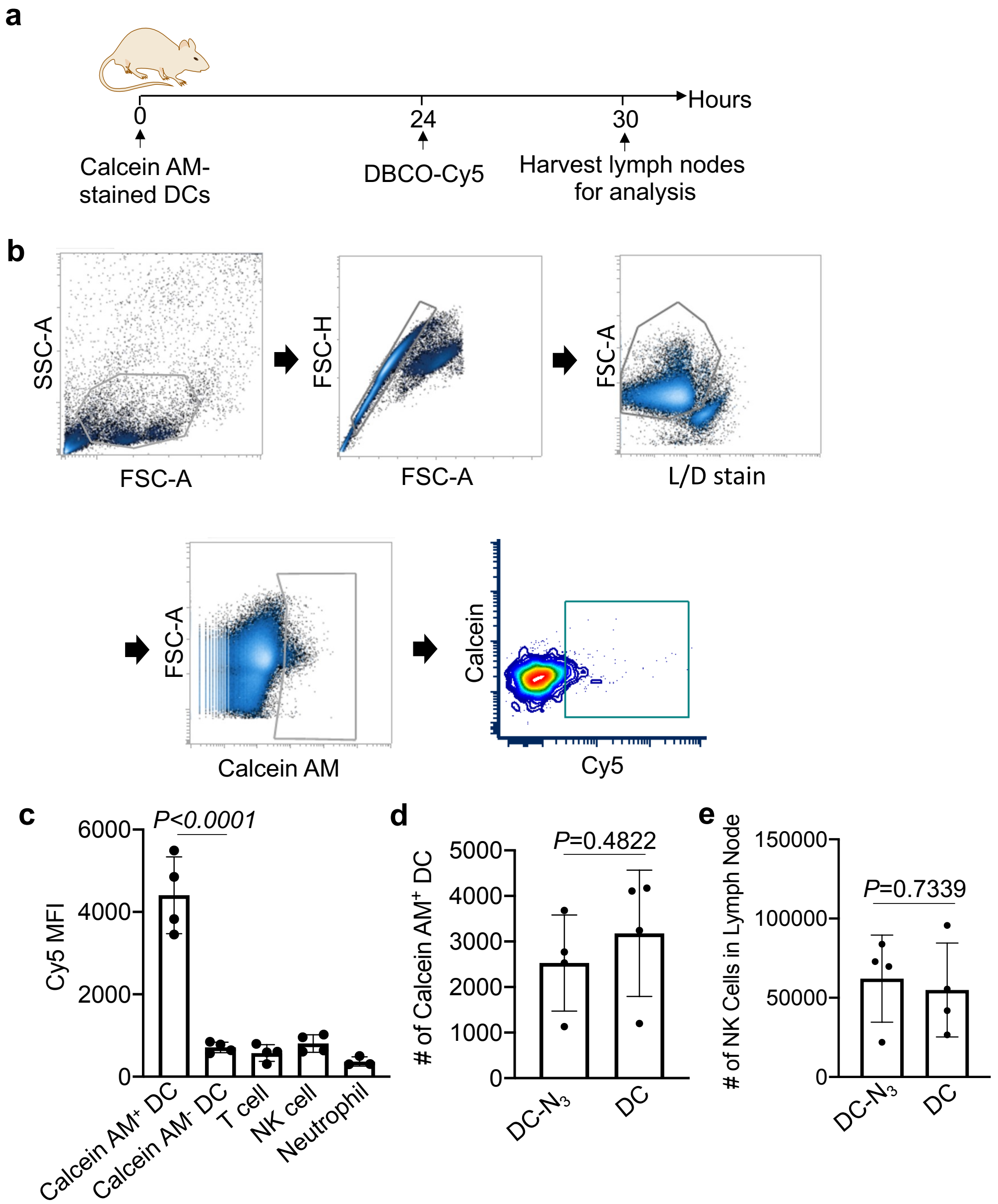

Supplementary Figure 14. *In vivo* conjugation of DBCO-Cy5 to adoptively transferred, azido-labeled DCs. (a) Timeframe of in vivo targeting study. DCs were pretreated with $Ac_4ManNAz$ or PBS for three days and stained with Calcein AM, prior to injection into C57BL/6 mice. DBCO-Cy5 was administered at 24 h, and lymph nodes were harvested for analysis at 30 h. (b) Representative gating strategy for analyzing $Cy5^{+}$Calcein AM^{+} cells. (c) Mean Cy5 fluorescence intensity of different immune cells in lymph nodes (n=4). (d) Number of Calcein AM^{+} DCs in the draining lymph nodes (n=4). (e) Number of natural killer cells in the draining lymph nodes (n=4). All the numerical data are presented as mean ± SD (two-tailed Welch's t-test was used; $0.01 < *P \leq 0.05$; $**P \leq 0.01$; $***P \leq 0.001$).

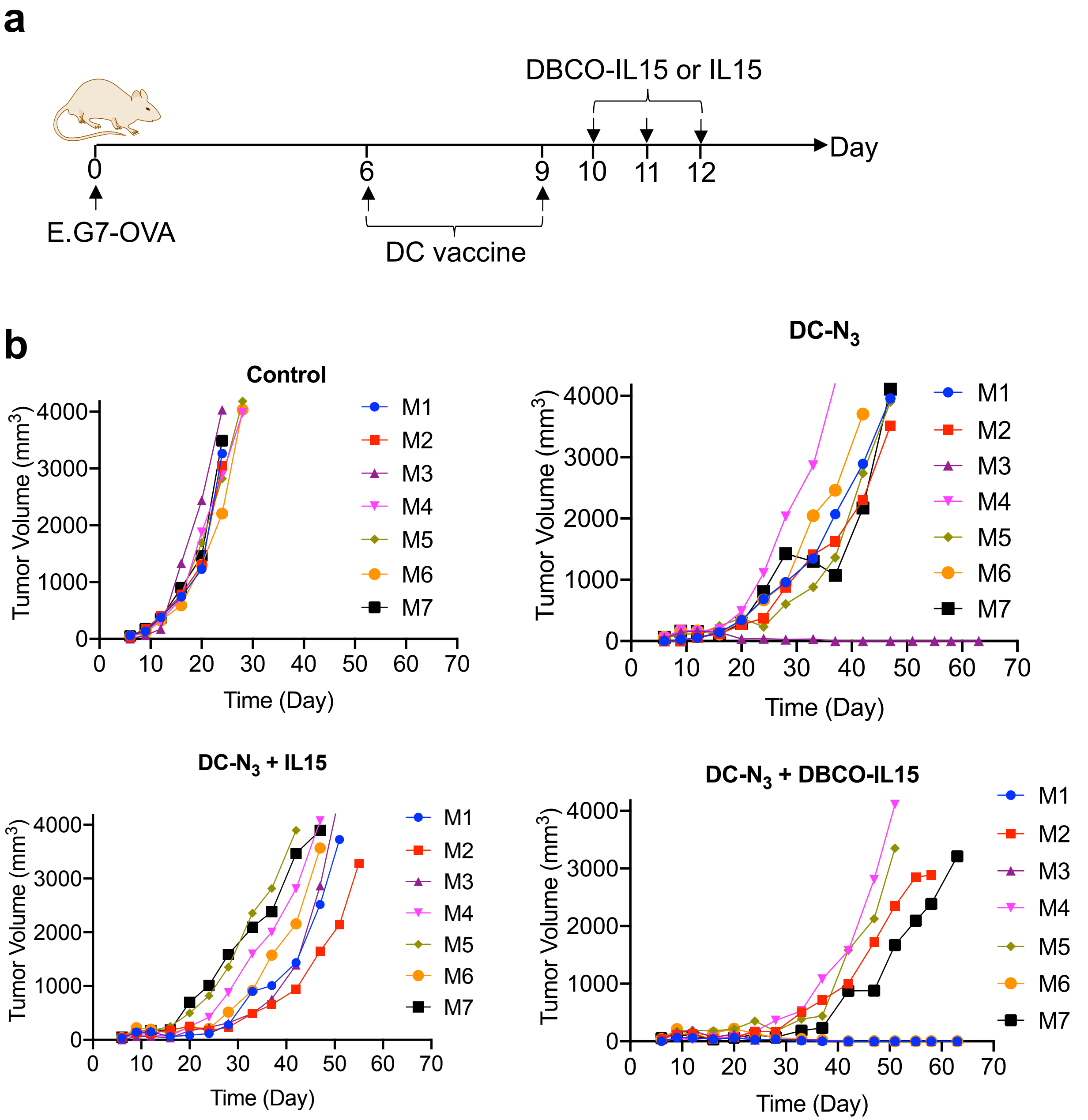

Supplementary Figure 15. ***In vivo*** **targeting of IL-15 onto adoptively transferred DC vaccines improves the antitumor efficacy.** (a) Timeframe of the therapeutic efficacy study. DCs were pretreated with $Ac_4ManNAz$ for three days and pulsed with SIINFEKL peptide for 16 h. (b) Tumor growth curves of individual mice in different groups.
